# Bifunctional Systems of *Amelanchier alnifolia* Leaves Extract-Oligosaccharides with Prebiotic and Antidiabetic Benefits

**DOI:** 10.3390/molecules30163327

**Published:** 2025-08-08

**Authors:** Anna Gościniak, Anna Sip, Piotr Szulc, Judyta Cielecka-Piontek

**Affiliations:** 1 Department of Pharmacognosy and Biomaterials, Poznan University of Medical Sciences, Rokietnicka 3, 60-806 Poznan, Poland; agosciniak@ump.edu.pl; 2 Department of Biotechnology and Food Microbiology, Poznań University of Life Sciences, Wojska Polskiego 48, 60-627 Poznan, Poland; anna.sip@up.poznan.pl; 3 Department of Agronomy, Poznan University of Life Sciences, Dojazd 11, 60-632 Poznan, Poland; piotr.szulc@up.poznan.pl

**Keywords:** prebiotics, oligosaccharides, plant extracts, microbiome, gut health

## Abstract

*Amelanchier alnifolia* is a plant known for its nutritional and bioactive properties. Its leaves contain a high concentration of active compounds with significant antioxidant and antidiabetic effects, including strong α-glucosidase inhibitory potential. The combination of these bioactive leaf extracts with prebiotic substances, such as fructooligosaccharides (FOS), galactooligosaccharides (GOS), and chitooligosaccharides (COS), enables the development of functional systems with enhanced beneficial properties. In this study, process optimization for leaves extraction was performed using a Plackett–Burman screening design, which identified key parameters for further optimization using the Box–Behnken design. The optimal extraction conditions were determined as follows: methanol content 58.06%, solid-to-solvent ratio 26.03 *m*/*v*, and extraction time 73.56 min. These conditions yielded the highest the total phenolic content (TPC). A comparative analysis of different cultivars revealed significant variations in polyphenol content among them. The formulated lyophilized systems with GOS, FOS and COS positively influenced the chlorogenic acid release profile, while maintaining the extract’s antidiabetic and antioxidant properties. FT-IR analysis confirmed the molecular interactions responsible for these effects. The prebiotic effectiveness of the systems was quantitatively evaluated using two key parameters: the prebiotic index (PI), and the prebiotic activity score (PAS). Microbiological analyses demonstrated the beneficial effects of prebiotic-enriched systems characterized by better prebiotic action on *Bifidobacterium* strains than the pure extract. These findings suggest that *A. alnifolia* leaf extracts, in combination with prebiotics, could serve as promising functional ingredients with potential applications in health-promoting and antidiabetic formulations.

## 1. Introduction

*Amelanchier alnifolia*, commonly referred to as the serviceberry or Saskatoon berry, is a deciduous shrub belonging to the *Rosaceae* family. This species is native to North America, particularly thriving in regions such as the southern Yukon, the Canadian Prairies, and parts of the northern United States. It has also been introduced in various parts of Europe due to its ornamental and edible qualities [1,2].

The leaves of *Amelanchier* species are known to contain a variety of bioactive compounds, particularly polyphenols from the group of flavonoids. These compounds are associated with antioxidant properties, which can help combat oxidative stress and inflammation in the body [3]. Studies have shown that the phytochemical composition *A. alnifolia* can be beneficial in promoting health, particularly in managing conditions related to metabolic syndrome and diabetes; however, most studies are focused on berries properties [4,5,6,7]. The antioxidant capacity of these leaves is attributed to the presence of flavonoids, which have been linked to various health-promoting effects, including anti-inflammatory and antidiabetic properties [3]. The genetic diversity within this genus allows for the selection of specific traits, such as fruit size and flavor, which can enhance both the esthetic and nutritional value of the plant [8,9]. Although the primary focus of *A. alnifolia* research has traditionally been on its fruits, our study emphasizes the leaves to explore their potential as a valuable source of bioactive compounds. Leaves often contain higher concentrations of certain phenolic compounds, such as flavonoids and chlorogenic acid, compared to fruits, making them a promising material for further investigation [10]. By analyzing the chemical composition of leaves, we aim to expand the understanding of their antioxidant and health-promoting properties. This approach not only complements fruit-based studies but also highlights the potential of utilizing leaves in functional food development or pharmaceutical applications.

Fructooligosaccharides (FOS), galactooligosaccharides (GOS), and chitooligosaccharides (COS) are functionally important oligosaccharides that have gained attention for their ability to modulate gut microbiota and support intestinal health. FOS and GOS are well-established and widely recognized prebiotics with documented effects on gut microbiota modulation and host health [11]. COS, while not yet officially classified as a prebiotic, demonstrates promising prebiotic-like properties and is increasingly studied as a functional oligosaccharide with both microbiota-stimulating and immunomodulatory effects [12,13,14,15]. These oligosaccharides are non-digestible carbohydrates that reach the colon intact, where they undergo fermentation by the gut microbiota, leading to the production of short-chain fatty acids (SCFAs) and other metabolites that confer health benefits [12,16,17].

FOS, which are composed of fructose units linked by β(2→1) glycosidic bonds, are primarily derived from plant sources such as chicory root and asparagus. Research has shown that FOS supplementation can significantly enhance the growth of beneficial bacteria, particularly *Bifidobacteria* and *Lactobacilli*, which are crucial for maintaining a healthy gut microbiome [18]. Similarly, GOS, which are derived from lactose and consist of galactose units linked by β(1→6) bonds, have been shown to exert prebiotic effects by promoting the growth of beneficial gut bacteria. GOS supplementation has been associated with improved gut barrier function, reduced intestinal permeability, and enhanced immune responses [19]. Studies indicate that GOS can effectively modulate gut microbiota composition, leading to favorable metabolic outcomes, including improved glycemic control and reduced inflammation [20]. COS also exhibit prebiotic properties. COS have been shown to stimulate the growth of beneficial gut bacteria and enhance SCFA production, similar to FOS and GOS [21]. Chitosan and chitooligosaccharides modulate the gut microbiome by promoting the growth of beneficial bacteria such as *Bifidobacteria* and *Lactobacillus*, improving gut health. Additionally, they regulate the microbial composition in obese and diabetic individuals by increasing bacteria associated with weight reduction and better glucose metabolism [22].

The health benefits of FOS, GOS, and COS extend beyond gut health. The SCFAs produced during the fermentation of these oligosaccharides play a crucial role in regulating various physiological processes, including appetite regulation, lipid metabolism, and immune function [23]. For instance, butyrate, one of the primary SCFAs, has been shown to have anti-inflammatory properties and may improve insulin sensitivity, making these oligosaccharides valuable components in dietary strategies aimed at preventing and managing metabolic diseases [24].

Moreover, the incorporation of FOS, GOS, and COS into functional foods and dietary supplements has gained traction in the food industry as a means to enhance gut health and provide additional health benefits. Their ability to improve the gut microbiome and metabolic health positions them as important ingredients in the development of health-promoting foods [25].

The combination of plant extracts, such as those derived from the leaves of *A. alnifolia*, with oligosaccharides like FOS, GOS, and COS, holds significant promise due to their dual-action potential. This synergy can enhance prebiotic effects by selectively fostering beneficial gut microbiota while simultaneously delivering bifunctional properties, such as prebiotic and antidiabetic effects. The integration of bioactive compounds from *A. alnifolia* leaves, rich in polyphenols, with oligosaccharides may amplify their individual health benefits, offering a comprehensive approach to improving gut health, modulating metabolic pathways, and managing conditions like diabetes.

The aim of this study was to develop bifunctional systems based on *A. alnifolia* leaf extracts combined with prebiotic oligosaccharides. The research included: optimization of extraction parameters using Plackett–Burman and Box–Behnken designs, HPLC profiling of phenolic compounds; assessment of antioxidant activity (CUPRAC, DPPH) and antidiabetic potential (α-glucosidase inhibition), principal component analysis (PCA) to correlate phenolic profiles with bioactivities, FT-IR analysis to identify molecular interactions with oligosaccharides, release studies of chlorogenic acid, and evaluation of prebiotic activity. These analyses allowed us to identify cultivars with the highest bioactive potential and to design stable, multifunctional systems with enhanced functional properties.

## 2. Results

### 2.1. Optimization of Extraction Conditions

#### 2.1.1. Optimization of Extraction Conditions Using Plackett–Burman Design

**Figure 1:**
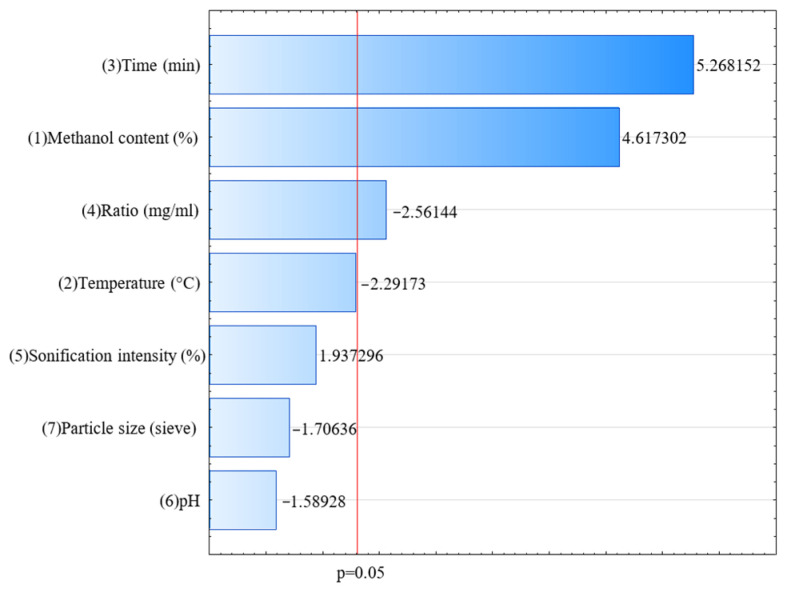
Pareto chart showing the effects of factors on total polyphenol content (TPC) extraction from *A. alnifolia* leaves (*p* < 0.05).

The optimization of bioactive compound extraction from *A. alnifolia* leaves was conducted with total polyphenol content (TPC) as the response variable, determined using the Folin–Ciocalteu method. The results, presented in the Pareto chart (Figure 1), revealed that extraction time and methanol content were the most influential factors, with standardized effects of 5.27 and 4.62, respectively, surpassing the significance threshold (*p* = 0.05). These parameters had a substantial positive impact on TPC, emphasizing their critical role in maximizing polyphenol extraction. The model showed a good fit, with a coefficient of determination (R^2^ = 0.89754) and an adjusted R^2^ = 0.80788. For further optimization of the extraction process, the parameters identified as significant in the Plackett–Burman design—namely extraction time, methanol content, and ratio—were selected.

#### 2.1.2. Optimization of Extraction Conditions Using Box–Behnken Design

**Figure 2:**
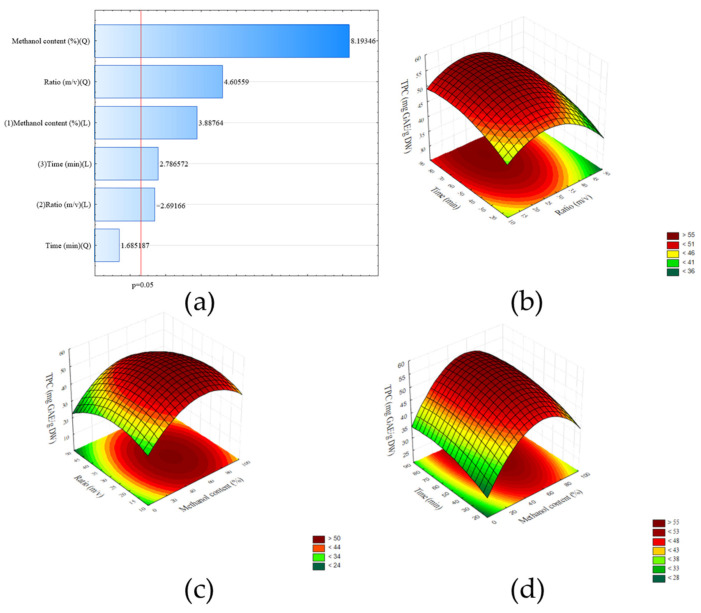
(**a**) Pareto chart showing the standardized effects of factors on total polyphenol content (TPC) in the Box–Behnken design (*p* < 0.05). Response surface plots illustrating the interactive effects of (**b**–**d**) methanol content, extraction time, and liquid-to-solid ratio on total polyphenol content (TPC) in the Box–Behnken design.

The optimization of the extraction process using the Box–Behnken design provided insights into the effects of key parameters—methanol content, extraction time, and liquid-to-solid ratio—on TPC. The Pareto chart (Figure 2a) identified methanol content as the most influential factor, with both its linear and quadratic effects significantly contributing to TPC. The liquid-to-solid ratio also showed a significant linear and quadratic effect, while extraction time exhibited a strong linear effect but no significant quadratic effect. The response surface plots (Figure 2b–d) illustrated the effects of these variables, showing that TPC increased with higher methanol content, longer extraction times, and moderate liquid-to-solid ratios.

The model suggested critical values for the parameters: methanol content of 58.06%, liquid-to-solid ratio of 26.03, and extraction time of 73.56 min, which were predicted to maximize TPC. The quality of the model was validated by a high coefficient of determination (R^2^ = 0.93429) and an adjusted R^2^ = 0.88501, indicating a strong fit between the experimental data and the model predictions. Based on the results, the parameters suggested by the model were applied to the extraction process (Table 1).

### 2.2. Cultivar Comparison

The leaf extracts from all cultivars were prepared under the optimized extraction conditions established in the earlier stage of the study. The eleven *A. alnifolia* cultivars exhibit distinct differences in their botanical traits, reflecting their diverse genetic and environmental adaptations. Northline, Pembina, and Sleyt demonstrate vigorous, spreading growth habits, whereas Obelisk, Thiessen, and Mandam exhibit upright or semi-upright growth forms. Fruit size varies from small to large, with morphologies ranging from round to oval and the consistent presence of a waxy bloom across all cultivars. Skin pigmentation includes hues from blue-black to purple-blue and occasionally red-purple. In terms of organoleptic properties, sweetness is most pronounced in Obelisk and Mandam, while higher acidity levels characterize Northline and Thiessen, with ripening phenology spanning from very early to late stages.

#### 2.2.1. TPC Comparison

**Figure 3:**
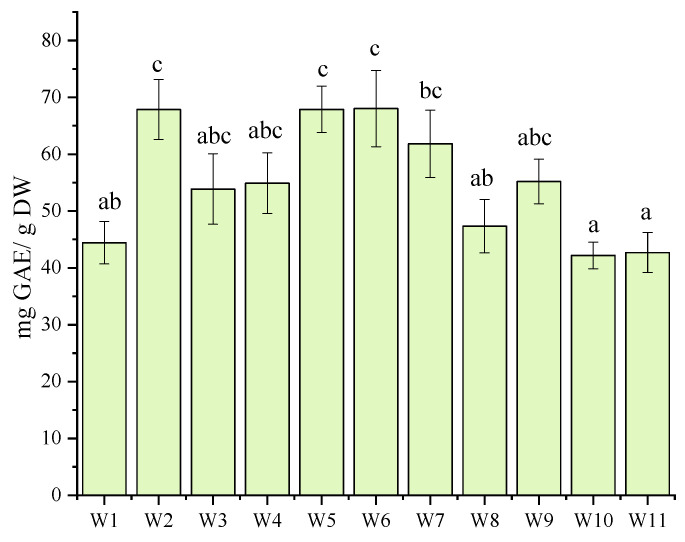
Total polyphenol content in *A. alnifolia* leaves cultivars measured using the Folin–Ciocalteu assay, expressed as Gallic acid equivalent (mg GAE/g DW). Bars marked with different letters differ significantly according to one-way ANOVA followed by Tukey’s post hoc test (*p* < 0.05).

The analysis revealed significant differences in polyphenol content among the studied cultivars. Some cultivars (W2–W7) exhibited notably higher levels, indicating greater bioactive potential. Others (W1, W9, W10, W11) showed lower polyphenol concentrations, which may affect their functional properties (Figure 3). These variations highlight the influence of genetic factors on polyphenol accumulation. Previous studies have shown that genotype strongly affects the biosynthesis of phenolic and flavonoid compounds, mainly through the regulation of enzymes in the phenylpropanoid pathway, such as phenylalanine ammonia-lyase (PAL) and chalcone synthase (CHS) [26,27]. Higher PAL activity has been associated with increased accumulation of flavonoids and anthocyanins, confirming that genetic background plays a critical role in determining both the content and the bioactivity of phenolic compounds.

#### 2.2.2. HPLC Analysis

**Figure 4:**
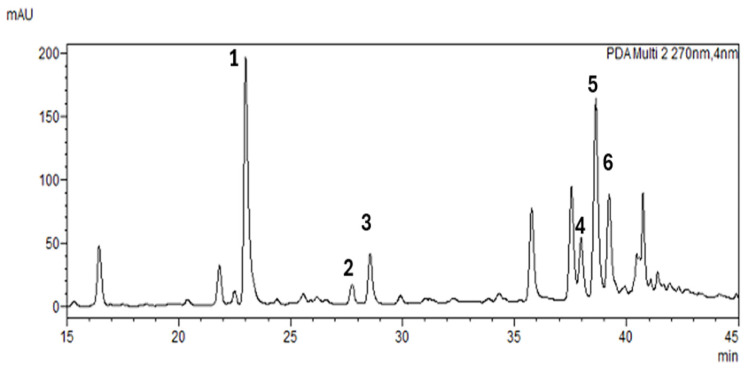
HPLC (λ  =  270 nm) fingerprint of the one of *A. alnifolia* leaves extract. Peaks were identified as follows: **1**—Chlorogenic acid, **2**—Epicatechin, **3**—Epigallocatechin gallate (EGCG), **4**—Rutin, **5**—Hyperoside, and **6**—Isoquercetin.

The HPLC analysis allowed for the quantification of key polyphenolic compounds across various *A. alnifolia* cultivars, providing a comprehensive fingerprint of their bioactive profiles (Figure 4). The quantified compounds included chlorogenic acid, epicatechin, EGCG, rutin, hyperoside, and isoquercetin, which are known for their antioxidant and potential health-promoting properties (Table 2). Among the analyzed samples, significant variations were observed in the concentration of these compounds. Chlorogenic acid was consistently the most abundant compound, with the highest content detected in sample W9 (19.38 ± 0.38 mg/g DW), while rutin and hyperoside showed notable variability, reaching peak concentrations in samples W6 (3.43 ± 0.04 mg/g DW) and W11 (6.35 ± 6.59 mg/g DW), respectively. Similarly, isoquercetin levels varied, with the highest content found in W11 (3.65 ± 0.15 mg/g DW). The study indicates that the tested cultivars differ in the content of active compounds, and not always one cultivar has the highest content of active compounds. However, the profile of each cultivar is different.

#### 2.2.3. Antioxidant Activity

**Figure 5:**
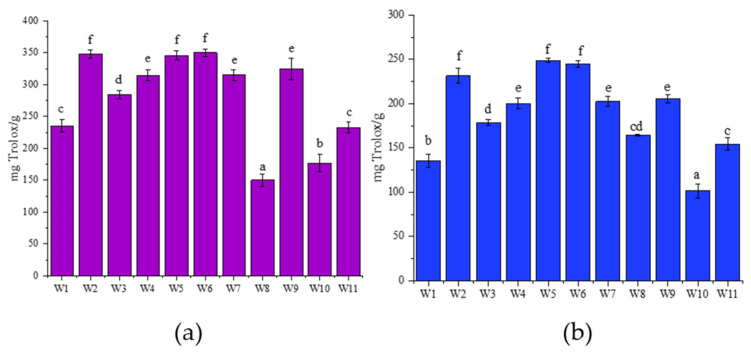
Antioxidant activity of *A. alnifolia* leaves cultivars measured using (**a**) the DPPH and (**b**) CUPRAC assay, expressed as Trolox equivalents (mg Trolox/g DW). Bars marked with different letters differ significantly according to one-way ANOVA followed by Tukey’s post hoc test (*p* < 0.05).

The antioxidant activity of *A. alnifolia* cultivars was assessed using the DPPH (Figure 5a) and CUPRAC (Figure 5b) methods. The results showed differences in antioxidant potential between the cultivars. In the CUPRAC assay, W9, W6, and W11 had the highest activity, reflecting their strong ability to reduce copper ions. In contrast, W10 and W1 showed the lowest activity. Similarly, in the DPPH assay, which measures free radical scavenging ability, W2, W5, and W6 again showed the highest activity, confirming their strong antioxidant potential. W8 and W10 had the lowest values, consistent with the CUPRAC results. These findings identify W2, W5, and W6 as cultivars with the strongest antioxidant activity and potential for further applications.

#### 2.2.4. Antidiabetic Activity

**Figure 6:**
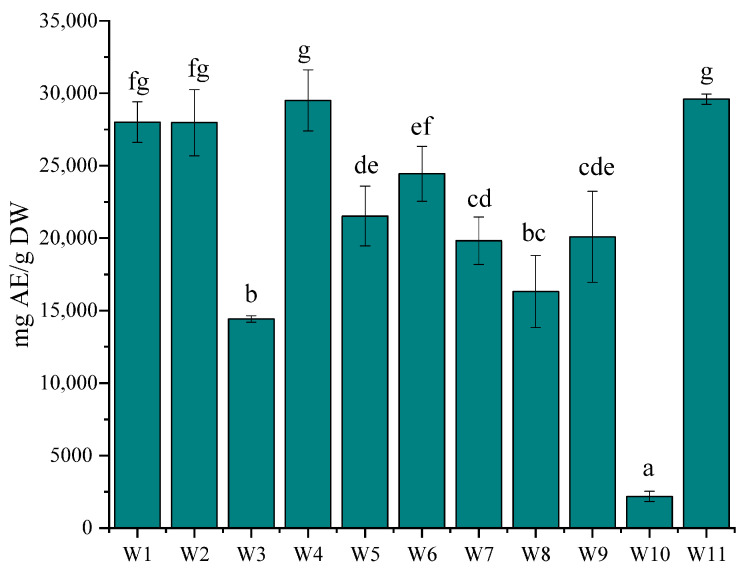
Antidiabetic activity of *A. alnifolia* leaves cultivars measured using the -glucosidase inhibition assay, expressed as acarbose equivalents (mg AE/g DW). Bars marked with different letters differ significantly according to one-way ANOVA followed by Tukey’s post hoc test (*p* < 0.05).

The antidiabetic activity of extracts from different *A. alnifolia* cultivars was assessed by their ability to inhibit the enzymes α-amylase and α-glucosidase (Figure 6). The results showed significant differences in the activity among the cultivars. The highest antidiabetic activity was observed in cultivar W1, W2, W4 and W11, which demonstrated significantly greater enzyme inhibition compared to the other cultivars. In contrast, the lowest activity was recorded for cultivar W10, suggesting a lower content of bioactive compounds responsible for enzyme inhibition.

### 2.3. Principal Component Analysis

**Figure 7:**
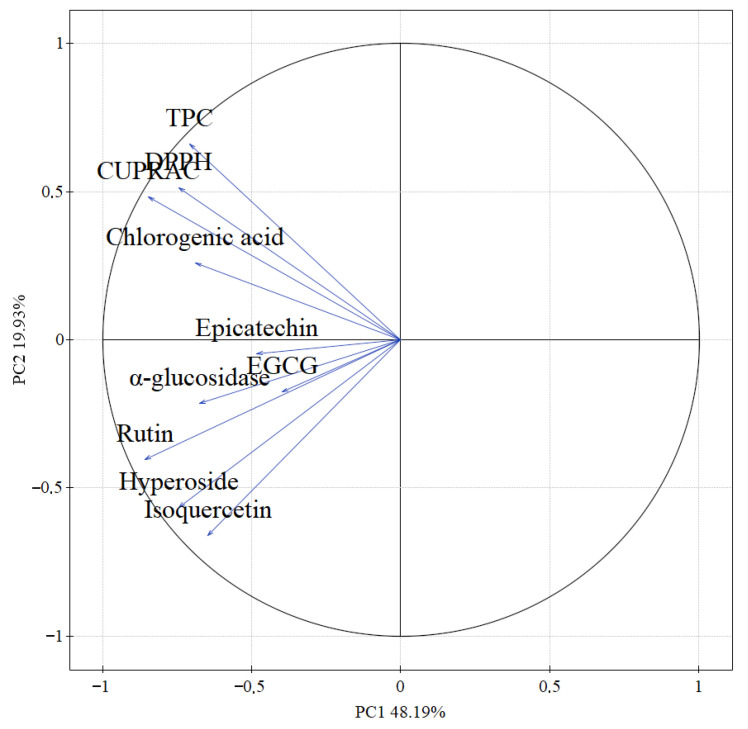
PCA plot of data from antioxidant activity, antidiabetic activity and content of active compounds.

The PCA reveals distinct relationships between polyphenolic compounds, antioxidant activity, and antidiabetic potential across the studied *A. alnifolia* cultivars (Figure 7). Total polyphenol content (TPC), CUPRAC, and DPPH are closely grouped and strongly correlated, indicating that higher polyphenol content directly contributes to increased antioxidant activity. Chlorogenic acid aligns closely with these variables, confirming its significant role in driving antioxidant capacity. In contrast, α-glucosidase inhibition shows a weaker correlation with TPC and antioxidant-related variables. Instead, it is more closely associated with specific compounds, such as hyperoside and isoquercetin, which are positioned in a different quadrant of the PCA plot. This separation suggests that antidiabetic activity depends more on the presence and concentrations of specific polyphenols rather than the overall polyphenol content.

### 2.4. System Obtaining and Assessing Properties

Based on a comprehensive analysis of total polyphenol content, antioxidant activity (CUPRAC and DPPH), and α-glucosidase inhibitory potential, cultivar W2 (Obelisk) was selected for further system development. This cultivar exhibited one of the highest levels of chlorogenic acid and a favorable profile of flavonoids, including rutin and hyperoside, which are strongly associated with antioxidant and antidiabetic effects. Moreover, W2 demonstrated consistently strong performance across multiple biological assays, indicating its superior bioactive potential compared to other cultivars. Therefore, it was considered the most promising candidate for incorporation into oligosaccharide-based delivery systems. Systems containing the lyophilized *A. alnifolia* leaves extract (Obelisk cultivar) and oligosaccharides (FOS, GOS, COS) were successfully obtained and subsequently ground into fine powders.

#### 2.4.1. FT-IR Analysis

**Figure 8:**
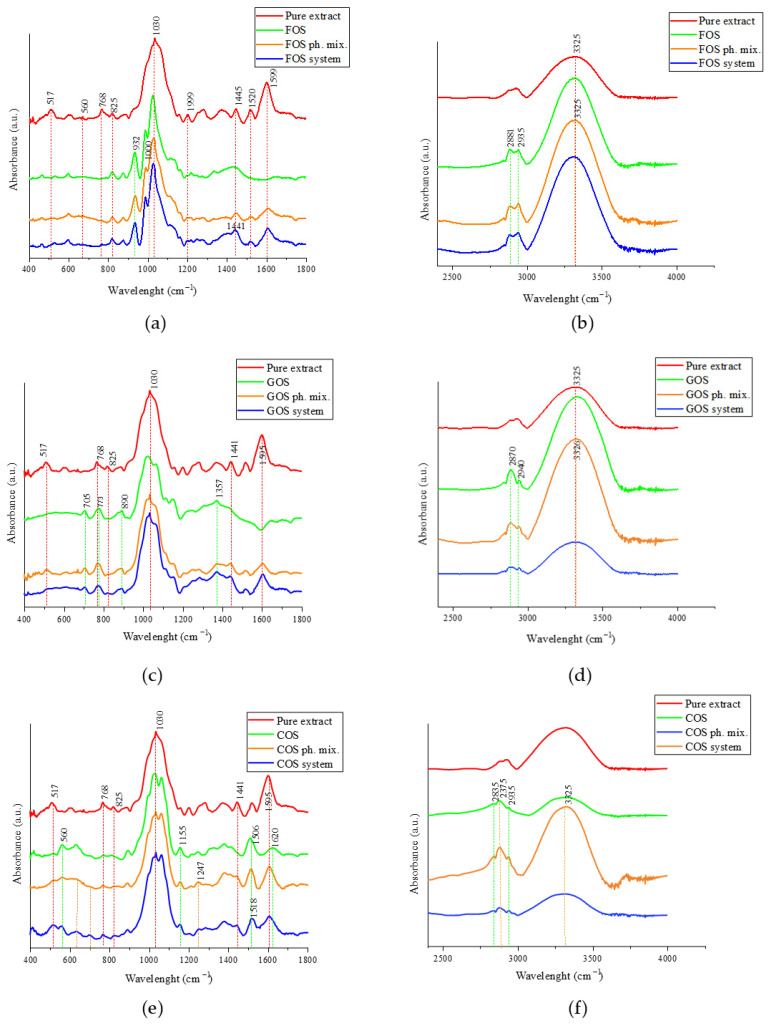
FT-IR spectra of *Amelanchier alnifolia* leaf extract (Pure extract, red) and systems with oligosaccharides: FOS, GOS, and COS. Spectra are shown for both physical mixtures (ph. mix.) and lyophilized formulations (system). Panels: (**a**,**b**) FOS-based systems; (**c**,**d**) GOS-based systems; (**e**,**f**) COS-based systems. Left panels (**a**,**c**,**e**) present the fingerprint region (400–1800 cm^−1^), while right panels (**b**,**d**,**f**) show the functional group region (2400–4000 cm^−1^).

FTIR analysis was performed to assess potential intermolecular interactions between Amelanchier leaves extract and oligosaccharides in the formulated systems. The FTIR spectrum of the freeze-dried *A. alnifolia* leaf extract displayed several characteristic absorption bands indicating the presence of polyphenols and glycosylated compounds (Figure 8). A strong and sharp peak at 1030 cm^−1^ corresponds to C–O stretching vibrations in alcohols, esters, and glycosidic bonds, indicative of flavonoid glycosides. The band at 1599 cm^−1^ is attributed to aromatic C=C stretching, typical for conjugated phenolic structures, while adjacent signals at 1520 cm^−1^ and 1445 cm^−1^ likely originate from skeletal vibrations of benzene rings and C–H bending modes, respectively [28]. In the lower wavenumber region, bands at 825 and 768 cm^−1^ can be assigned to out-of-plane C–H bending of substituted aromatic rings, supporting the presence of polyhydroxylated flavonoids [29]. The band at 560 cm^−1^ is associated with ring deformations, while 517 cm^−1^ likely results from skeletal vibrations in aromatic systems [30]. Collectively, these absorption features confirm the polyphenol-rich profile of the extract, consistent with the presence of chlorogenic acid, rutin, and isoquercetin as identified by HPLC. A broad and intense O–H stretching vibration was observed at 3325 cm^−1^, confirming extensive hydroxyl functionalities typical of phenolic acids and flavonoids [31].

The FTIR analysis revealed distinct spectral differences between the physical mixtures and lyophilized systems across all prebiotic carriers (FOS, GOS, and COS). The FT-IR spectra of the pure extract, FOS, physical mixture, and the lyophilized FOS system revealed characteristic features corresponding to both components, suggesting the formation of non-covalent interactions rather than chemical modifications (Figure 8a,b). In the fingerprint region (Figure 8a), FOS exhibited strong absorption bands at 932 cm^−1^ and 1000 cm^−1^, corresponding to C–O and C–C stretching vibrations typical of oligosaccharides [32]. These peaks were preserved in both the physical mixture and the lyophilized system, indicating the structural integrity of FOS. Additionally, a slight shift in the band at 1445 cm^−1^ in the pure extract to 1441 cm^−1^ in the FOS-containing systems was observed, suggesting possible hydrogen bonding or dipole–dipole interactions between the polyphenols and the saccharide matrix. Several bands in the extract spectrum, including, 560 cm^−1^, 768 cm^−1^, 825 cm^−1^, 1030 cm^−1^, 1520 cm^−1^, and 1599 cm^−1^, were associated with aromatic ring deformations, C–H bending, and C=C stretching of polyphenolic compounds. These peaks appeared to decrease in intensity or become overlapped in the formulation spectra. In the higher wavenumber region (Figure 8b), a broad absorption band centered at 3325 cm^−1^ was attributed to O–H stretching vibrations, typical for hydroxyl-rich compounds involved in hydrogen bonding [33]. This band was present in all samples but showed reduced intensity in the lyophilized system, suggesting altered hydrogen bond dynamics upon formulation. The bands at 2870 cm^−1^ and 2935 cm^−1^, corresponding to C–H stretching in aliphatic chains, remained visible in all spectra and confirmed the presence of saccharide structures [34]. Overall, the spectra indicate that the lyophilization process led to mild intermolecular interactions between FOS and extract components, mainly via hydrogen bonding, while preserving the main structural features of both constituents. The observed spectral features result from the superposition of signals from individual components with minor shifts indicative of non-covalent binding.

The FT-IR spectra of the pure extract, GOS, physical mixture, and GOS-based system (Figure 8c,d) demonstrate both additive and interactive spectral characteristics. In the GOS system, the 517 cm^−1^ band is diminished compared to the physical mixture, indicating a potential loss of structural freedom or masking due to matrix entrapment. The most intense peak at 1030 cm^−1^, corresponding to C–O–C stretching in both extract and GOS, is retained but shows slight broadening in the system, suggesting overlapping contributions [35]. Bands at 768 and 1357 cm^−1^ exhibit shape deformation or widening, which may be attributed to altered hydrogen bonding or conformational changes. Crucially, a shift in the 1595 cm^−1^ band is observed in the system, supporting the formation of intermolecular interactions, most likely hydrogen bonds, between GOS and active extract components. In the higher wavenumber region (Figure 8d), the broad O–H stretching band at 3325 cm^−1^ becomes wider and less intense in the GOS system, indicating more extensive hydrogen bonding. Additionally, the bands at 2870 cm^−1^ and 2940 cm^−1^ (C–H stretching) are slightly altered in intensity, which may also reflect matrix-related conformational effects.

The FT-IR spectra of the pure extract, COS, physical mixture, and COS-based system (Figure 8d–f) reveal characteristic changes indicating molecular-level interactions between extract components and the carrier matrix. In the COS-based system, significant modifications are observed: the 517 cm^−1^ peak undergoes shape broadening, suggesting overlapping or restricted vibrational freedom. The 1155 cm^−1^ and 1247 cm^−1^ bands are also deformed in the system, which may indicate interactions involving ether or glycosidic linkages [36]. A notable shift in the 1506 cm^−1^ band to 1518 cm^−1^ is observed in the system spectrum, suggesting the formation of specific hydrogen bonding or dipole interactions between extract polyphenols and COS [37]. The preservation but broadening of the intense 1030 cm^−1^ band reflects band superposition from both extract and COS. In the higher wavenumber region (Figure 8f), the broad O–H stretching band at 3325 cm^−1^ appears flattened and less intense in the COS system compared to the pure extract, consistent with stronger or more numerous hydrogen bonds. Additionally, bands in the ~2885–2935 cm^−1^ region (C–H stretching) show minor positional and intensity changes, likely reflecting matrix packing effects [38]. Overall, the FT-IR profile of the COS system reflects overlapping spectral features with clear band deformation and shifts, indicating interactions between COS and bioactive components of the extract, particularly via hydrogen bonding and physical entrapment within the matrix [34,39].

#### 2.4.2. Content of Active Compounds

The content of active compounds was analyzed in the lyophilizate obtained from the W2 extract and in the systems with oligosaccharides (Table 3). It was found that the content of active compounds in the systems was twice as low as in the lyophilizate, indicating that the lyophilization process and formulation development did not affect the active compound content.

#### 2.4.3. Antioxidant Activity of Obtained Systems

As shown in Table 4, the antioxidant activity of the formulated systems was approximately two-fold lower than that of the pure extract (*p* < 0.05), which is consistent with the 1:1 dilution of bioactive compounds in the systems. All systems differed significantly from ascorbic acid and the pure extract, while no significant differences were observed between the FOS, GOS and COS systems. Despite the preserved proportional activity, the systems did not demonstrate additive or synergistic biological effects, indicating that the added oligosaccharides served more as carriers than enhancers of antioxidative function. These observations were confirmed using both the CUPRAC and DPPH methods, demonstrating consistency in the antioxidant activity results across different analytical techniques (Table 4). The activity of the obtained systems is comparable to that of ascorbic acid, highlighting the strong antioxidant activity of Amelanchier leaves and, consequently, the formulations derived from them.

#### 2.4.4. Antidiabetic Activity of Obtained Systems

The study showed that in the case of FOS and GOS, the antidiabetic activity, assessed through α-glucosidase inhibition, was approximately two times lower than that of the lyophilizate, indicating that the activity remained unchanged, as the systems were prepared in a 1:1 ratio (Table 5). Although chitooligosaccharides (COS) are reported in the literature to have antidiabetic properties, no significant α-glucosidase inhibitory activity was observed at the tested concentrations [40]. This suggests that the observed activity in COS systems was entirely due to the plant extract. The observed differences resulted solely from the 1:1 ratio, confirming that the antidiabetic activity was preserved. Compared to acarbose, a reference antidiabetic drug with the same mechanism of action, the antidiabetic activity of the systems is approximately 10 times higher. This demonstrates the strong α-glucosidase inhibitory potential of Amelanchier leaves extracts and the effectiveness of the developed formulations in inhibiting carbohydrate digestion, which could be beneficial for blood sugar regulation.

#### 2.4.5. PAMPA Assay

The permeability of the tested samples was evaluated using the PAMPA assay, with results expressed as apparent permeability coefficient (P_app_). (Table 6) All analyzed samples, including the FOS system, GOS system, COS system, and lyophilizate, exhibited permeability values below 1 × 10^−6^ cm/s. These results indicate that the samples have low passive permeability across the artificial membrane, classifying them as poorly permeable compounds. This suggests limited absorption potential via passive diffusion under the tested conditions. This limited permeability may be advantageous, as it increases the likelihood that the compounds reach the intestinal lumen in an unabsorbed form, where they can exert local effects such as α-glucosidase inhibition at the epithelial surface and modulation of gut microbiota, thereby contributing to both antidiabetic and prebiotic activities.

#### 2.4.6. Dissolution Study

**Figure 9:**
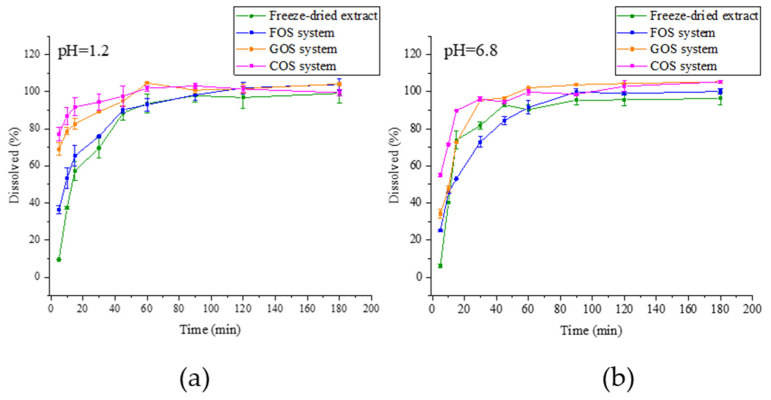
Chlorogenic acid release profile from freeze-dried Amelanchier leaf extract and systems obtained with FOS, GOS, and COS at pH 1.2 (**a**) and pH 6.8 (**b**).

The release profiles of bioactive compounds from the lyophilized extract, FOS system, GOS system, and COS system show clear differences (Figure 9). All systems demonstrated a rapid initial release, followed by a plateau phase. The lyophilized extract showed the slowest release in the initial phase, indicating a more gradual release compared to the other systems. The COS system had the fastest initial release, reaching a plateau earlier. The GOS and FOS systems displayed intermediate release rates, with similar patterns over time. These results suggest that the type of system affects the release behavior, with the COS system providing faster release, while the lyophilized extract offers a more sustained release profile.

#### 2.4.7. Microbiological Study

**Figure 10:**
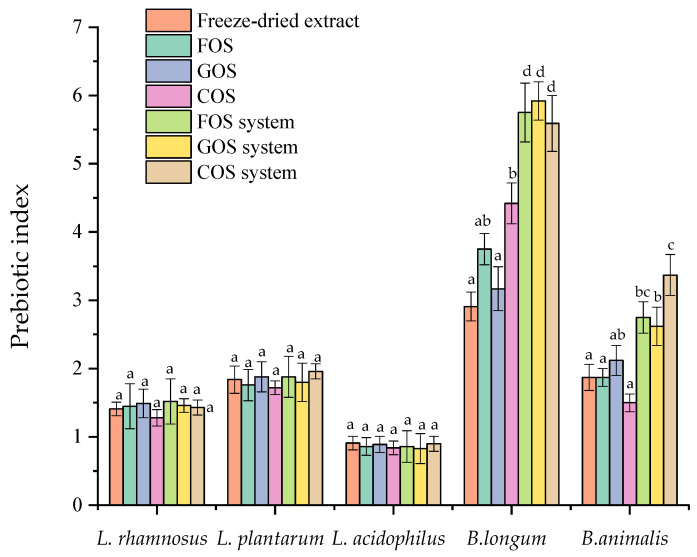
Prebiotic index values of probiotic strains of *Lactobacillus* and *Bifidobacterium* grown in different prebiotic systems and pure extract. Error bars are ± SD. Bars marked with different superscript letters differ significantly (*p* < 0.05, one-way ANOVA followed by Tukey’s post hoc test).

**Figure 11:**
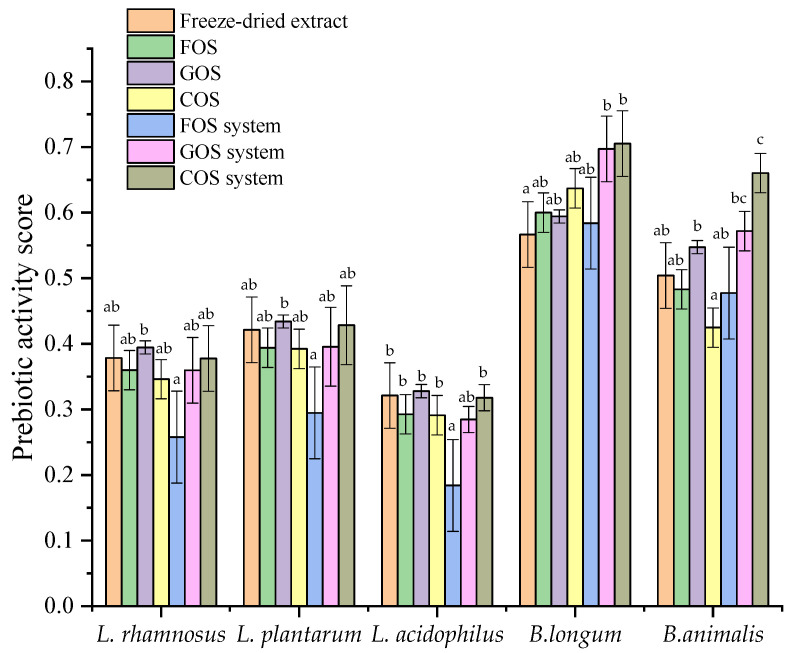
Prebiotic activity scores of different prebiotic systems and pure extract. Error bars are ± SD. Bars marked with different superscript letters differ significantly (*p* < 0.05, one-way ANOVA followed by Tukey’s post hoc test).

The data in Figure 10 show that, the tested plant extract significantly promoted the growth of most analyzed probiotic strains, especially those belonging to the genus *Bifidobacterium*, as reflected by a prebiotic index (PI > 1). Such an effect was not found only in the case of the *L. acidophilus*. The most pronounced growth stimulation was observed for *B. longum* and *B. animalis*, suggesting that *Bifidobacterium* species are more responsive by the extract. Moreover, the extract exhibited effects comparable to established prebiotics such as FOS, GOS, and COS. The highest prebiotic index (PI) values were recorded for the formulated multi-component systems (FOS system, GOS system, COS system), indicating a synergistic effect of their components. For *B. longum*, the PI of these systems was 1.5 to 2 times higher than that of the individual components, highlighting the value of combining oligosaccharides to enhance their prebiotic properties. Furthermore, the prebiotic activity scores (Figure 11) confirmed the selective action of the tested compounds. All analyzed prebiotics and the extract promoted the growth of probiotic strains and at the same time did not affect the growth of pathogenic *E. coli*, which is a domain true prebiotic activity. The highest PAS values were again observed for B. longum, reinforcing its susceptibility to the prebiotic systems. However, it is worth noting that the FOS-based system (FOS system) showed a lower prebiotic index than FOS alone, which may indicate that this combination could also support the growth of non-beneficial bacteria such as *E. coli.* Therefore, despite its stimulatory effect on probiotics, the prebiotic suitability of this system should be verified in further in vivo studies.

## 3. Discussion

Optimization methods, such as the Plackett–Burman and Box–Behnken designs, are extensively utilized in the field of natural product research to determine the most effective extraction parameters for bioactive compounds. These methodologies enable systematic evaluation of various factors, allowing researchers to develop efficient, reproducible, and scalable extraction protocols. In this study, these approaches were employed to optimize the extraction of polyphenols from *A. alnifolia* leaves—a relatively unexplored matrix in terms of bioactive compound recovery. The optimized extraction parameters—methanol content of 58.06%, solvent-to-solid ratio of 26.03 mL/g, and extraction time of 73.56 min—align with conditions reported for other polyphenol-rich plant matrices. Research on *Vaccinium myrtillus* leaves highlighted the efficacy of ethanol solutions (30%) and specific extraction durations of 5 min in the sonotrode extraction method [41]. Similarly, a study on *Moringa oleifera* leaves demonstrated the effectiveness of 80% ethanol and moderate extraction conditions (50 °C, 60 min), using a solvent-to-solid ratio of 60:1 (*v*/*w*), leading to high phenolic yields and significant antioxidant activity [42]. Optimization using ultrasound-assisted extraction of olive leaves revealed that solvent composition, extraction time, and liquid-to-solid ratio are critical parameters for polyphenol recovery. These optimized conditions (120 min, 25 °C, 70% ethanol) utilized a solvent-to-solid ratio of 5:1 (*v*/*w*), resulting in extracts with enhanced antioxidant properties [43]. While the fruits of *A. alnifolia* have been extensively studied for their polyphenol content and associated health benefits, the leaves represent an underutilized resource with significant potential. An optimization technique was applied to the drying process of Saskatoon berries. In the study by Mitra and Meda [44], the authors optimized the microwave–vacuum drying process for *A. alnifolia* berries. The study employed response surface methodology to determine optimal drying parameters, such as microwave power, drying time, and sample load, to achieve the highest product quality.

The observed differences in botanical traits among the *A. alnifolia* cultivars, including growth habit, fruit size, shape, and chemical composition, highlight the genetic diversity within the species. These differences may play a crucial role in determining their adaptability to specific environmental conditions and their suitability for commercial cultivation, particularly for producing fruits with valuable health-promoting nutritional properties that can benefit human health. In our study, leaves of various cultivars were compared in terms of their antioxidant activity, antidiabetic potential, and the content of bioactive compounds. The analysis revealed statistically significant differences between cultivars; however, it is important to note that the study was limited to samples collected from a single growing season. The results highlighted variability in the levels of active compounds across cultivars, consistent with findings from other species. For instance, Zhang et al. [45] compared the phytochemical profiles and antioxidant capacities of olive leaves (*Olea europaea* L.) from 32 cultivars grown in China, finding significant differences. Amelanchier research often focuses on the differences between species in the genus. Anatomical studies on Amelanchier species, both in vitro and ex vitro, indicated no differences in the stomatal apparatus of plantlets, with hypostomatic leaf characteristics observed in both ‘Krasnoyarskaya’ (*A. alnifolia*) and ‘Prince William’ (*A. canadensis*). Didur et al. [46] reported variability in secondary metabolite accumulation and antioxidant properties among Amelanchier species, with *A. humilis* exhibiting the highest antioxidant potential. But the differences between varieties of *A. alnifolia* were also studied. Research by Szpadzik and Krupa, conducted under the climatic and soil conditions of eastern Poland, evaluated three Canadian Saskatoon berry cultivars (‘Honeywood’, ‘Martin’, and ‘Pembina’), revealing significant differences in yield, fruit physicochemical properties (mass, diameter, firmness, soluble solids content), and health-promoting compounds (anthocyanins, flavonoids, polyphenols) [47].

In this study, the content of selected bioactive compounds in the leaves of *A. alnifolia* was analyzed. The results demonstrated the high content of chlorogenic acid, epicatechin, EGCG, rutin, hyperoside, and isoquercetin across the samples (W1–W11). Lavola et al. [48] reported that the leaves of various Amelanchier cultivars contained high amounts of quercetin- and kaempferol-derived glycosides (41% of total phenolics), hydroxycinnamic acids (36%), catechins, and some neolignans, with quercetin 3-galactoside, quercetin 3-glucoside, (−)-epicatechin, and chlorogenic acid as the dominant phenolics. These findings confirm the richness of Amelanchier species in phenolic compounds. Furthermore, Meczarska et al. [49] determined the polyphenolic composition and biological activity of extracts from both fruits and leaves of *A. alnifolia*. Their results showed that the tested extracts are rich sources of polyphenols, primarily flavonoids, with flavonols and anthocyanins dominating in leaves and fruits, respectively. Their study also confirmed high levels of chlorogenic acid and glycosidic derivatives of quercetin, which are consistent with the findings in this study. Lachowicz et al. [50] investigated the composition of bioactive compounds and antioxidant activity in the fruits of different genotypes of *A. alnifolia* cultivated in central Poland. They confirmed thirty-eight bioactive compounds, including polyphenolic compounds (four anthocyanins, nine phenolic acids, nine flavonols, and seven flavan-3-ols), carotenoids, chlorophylls, and triterpenoids, confirming that the variety has a significant effect on the content of active compounds.

The antioxidant activity of plant materials is closely linked to their polyphenolic content, as polyphenols are well-known for their ability to neutralize free radicals and reduce oxidative stress. The antioxidant activity demonstrated in our study indicates a high antioxidant potential of the examined cultivars. So far, the antioxidant activity of berries has been quantified in various studies, revealing a range of antioxidant capacities. For instance, Asyakina et al. [51] reported that the antioxidant activity of different Saskatoon berry varieties ranged from 8.68 to 35.66 mmol/100 g in Trolox equivalents, with an average of 23.9 mmol/100 g against DPPH radicals Similarly, Lachowicz et al. [50] found that the antioxidant capacity of Saskatoon berries correlated positively with their phenolic content, including flavanols and procyanidins, suggesting that the chemical composition significantly influences their antioxidant potential Lachowicz et al. reported that the antioxidant activity of Saskatoon berry extracts reached values of 13.09, 3.04, and 21.00 µmol Trolox/g d.s ABTS, DPPH, and FRAP tests, respectively. Antioxidant activity is critical because it plays a key role in protecting cells from oxidative damage, which is associated with chronic diseases such as cardiovascular disease, diabetes, and cancer [52]. By mitigating oxidative stress, antioxidants contribute to overall cellular health and help maintain physiological balance, emphasizing the potential of these cultivars as sources of natural antioxidants for use in functional foods, dietary supplements, and pharmaceuticals [53].

Our study demonstrated that the *A. alnifolia* cultivars exhibit antidiabetic activity through their ability to inhibit α-glucosidase in vitro. Significant differences in the inhibitory activity were observed among the cultivars, reflecting variability in their bioactive compound profiles. Previous studies have primarily focused on the antidiabetic properties of *A. alnifolia* fruits, demonstrating their potential in modulating glucose metabolism. Our findings expand this knowledge by confirming that different cultivars vary significantly in their inhibitory effects, highlighting their potential for functional food applications. In a study examining the metabolic effects of Saskatoon berries, it was found that they regulate glucose metabolism and improve cardiovascular health in models of diet-induced metabolic syndrome [54]. The non-polar extracts of Saskatoon berries have been shown to inhibit aldose reductase, an enzyme implicated in diabetic complications, with an inhibition rate of 82% [3]. This suggests that Saskatoon berries may play a role in preventing diabetic microvascular complications by modulating enzyme activity related to glucose metabolism. In vitro studies have indicated that extracts from Saskatoon berries also can significantly inhibit α-glucosidase activity [5,55]. This effect is attributed to the presence of various bioactive compounds, including anthocyanins and other polyphenolic compounds, which have been recognized for their potential to modulate carbohydrate metabolism [56].

Principal component analysis (PCA) showed a strong association between antioxidant and antidiabetic activity and polyphenol content. The results show that parameters such as DPPH, CUPRAC, and total polyphenol content (TPC) are strongly correlated, suggesting that the higher the content of these compounds, the greater the antioxidant potential of the extracts tested. In particular, chlorogenic acid and epicatechin show a positive correlation with antioxidant activity, which is in line with previous studies on their role as effective free radical scavengers [57,58,59]. Higher concentrations of phenolic compounds are often correlated with greater antioxidant potential, highlighting the importance of these compounds in evaluating the health-promoting properties of plants. Furthermore, PCA indicates that antidiabetic activity, as measured by α-glucosidase inhibition, is correlated with the presence of flavonoids such as rutin, hyperoside, and isoquercetin. This is consistent with studies on flavonoids, which showed the ability to inhibit the enzymes responsible for breaking down carbohydrates and reducing blood glucose spikes [60,61,62].

Our study aimed to obtain systems of extract and oligosaccharides through lyophilization. Lyophilization is a technique that enables the formation of stable systems by removing water through sublimation, preserving the structural and functional properties of the components. FT-IR spectroscopy revealed notable spectral modifications upon formulation of Amelanchier leaves extract with oligosaccharide carriers (FOS, GOS, COS). All systems showed overlapping contributions from both the extract and the polysaccharide matrix; however, subtle shifts and band distortions were evident, indicating weak intermolecular interactions. Among the formulations, the GOS and COS systems exhibited more pronounced spectral changes compared to FOS. Specifically, the COS system showed a visible broadening or deformation of bands at 517, 1155, and 1247 cm^−1^, as well as a shift in the aromatic skeletal vibration from 1506 to 1518 cm^−1^. These changes suggest hydrogen bonding between phenolic hydroxyl groups and the chito-oligosaccharide network. The O–H stretching region (~3325 cm^−1^) also became broader and less intense in the COS and GOS systems, reflecting alterations in hydrogen bond dynamics or increased network complexity. The FOS system, in contrast, showed relatively minor modifications, such as a small shift in the 1445 cm^−1^ band to 1441 cm^−1^, implying weaker interactions. These findings indicate that the strength and extent of interaction between extract constituents and carrier matrices vary depending on oligosaccharide type, with COS showing the strongest evidence of matrix–polyphenol interaction. Comparable FT-IR results have been reported in the literature for phenolic compound encapsulation. For example, Peng et al. [63] described shifts in the aromatic C=C and C–O stretching regions upon the interaction of flavonoids with polysaccharides, confirming that non-covalent interactions such as hydrogen bonding play a key role in system stability and compound retention.

The study demonstrated that the content of bioactive compounds, as well as the antioxidant and antidiabetic activities, remained unchanged after the incorporation of oligosaccharides FOS, GOS, or COS into the system. In the case of COS, the antioxidant activity did not increase; however, COS exhibits its intrinsic antioxidant properties, which have also been reported in other studies [64,65]. Nevertheless, its activity is significantly lower than required to be noticeable at the studied concentrations. Despite literature reports on the antidiabetic effects of COS through glycosidase inhibition, no increase in antidiabetic activity was observed in the systems containing these oligosaccharides. This is likely due to the low concentrations of the system used in the test as a result of the strong activity of the *A. alnifolia* leaf extract. It is worth mentioning that the antidiabetic activity of COS is widely documented in the literature. For instance, Makalani et al. reported that COS improved serum biochemical markers and histological changes in the pancreas of streptozotocin (STZ)-induced diabetic rats, suggesting a long-term antidiabetic effect through enhanced glucose metabolism and pancreatic secretory capacity [66]. This aligns with findings from Liu et al., who noted that low molecular weight chitosan could stimulate glucagon-like peptide-1 (GLP-1) secretion in human intestinal endocrine cells, a hormone crucial for glucose regulation [67]. Additionally, numerous studies on oligosaccharides (OS) highlight their prebiotic activity and their associated role in diabetes regulation [68,69,70,71].

In the conducted study, it was observed that compounds present in *A. alnifolia* leaves did not permeate through the membrane in the PAMPA assay, indicating limited passive permeability across biological membranes. This limited permeability may be attributed to their chemical properties, such as high polarity and the presence of multiple hydroxyl groups, which hinder diffusion through the hydrophobic lipid layers of membranes. This low permeability was also confirmed in the study by Petit et al. [72]. Low permeability of compounds in the small intestine may not necessarily be detrimental to α-glucosidase inhibition. For instance, certain polyphenolic compounds, such as chlorogenic acid, have been shown to possess inhibitory effects on α-glucosidase despite their varying permeability profiles. Chlorogenic acid, in particular, has demonstrated significant inhibitory activity against α-glucosidase, with studies indicating that it can reduce enzyme activity by forming complexes with the enzyme [73,74]. Once ingested, many of these compounds are not absorbed in the small intestine due to their high molecular weight and low solubility in water. Instead, they reach the large intestine, where the intestinal microflora metabolizes them. In the large intestine, polyphenols are fermented by different strains of bacteria, leading to the formation of various metabolites such as organic acids, phenols and other bioactive compounds. These metabolites may have beneficial health effects, including anti-inflammatory, antioxidant, and gut health-promoting effects [75,76].

Dissolution studies have demonstrated that the incorporation of oligosaccharides significantly enhances the solubility of chlorogenic acid in extracts from Saskatoon leaves. This improvement suggests that oligosaccharides may facilitate the bioavailability of chlorogenic acid in aqueous systems, which could be particularly advantageous for the development of functional formulations. Such a beneficial effect was also achieved in Stasilowicz-Krzemień et al., where rosmarinic acid was combined with cyclodextrins, and a release profile with a faster release in the first minutes was obtained [77]. The results of the release study correlate with the FT-IR data. The system with COS, which showed the strongest changes in the spectrum suggesting the appearance of new bonds, showed the greatest improvements in solubility, while the system with FOS showed the least. The observed increase in solubility may be attributed to molecular interactions, such as hydrogen bonding or encapsulation mechanisms, which enhance the dispersion of chlorogenic acid in the extraction medium.

The results of the prebiotic activity study showed that Amelanchier leaves extract effectively improves the growth of prebiotic bacteria, especially for *Bifidobacterium animali* and *Bifidobacterium longum*. Particularly noteworthy are the findings related to the formulated multi-component systems (FOS, GOS, and COS systems), which showed the highest values for both the prebiotic index and the prebiotic activity score. The observed synergy between the system components may result from complementary metabolic pathways or differential affinities to bacterial transport systems. Research indicates that the polyphenolic compounds in these berries may influence gut microbiota composition, promoting beneficial bacteria. For instance, a study found that Amelanchier berry powder supplementation in high-fat, high-sucrose diet-induced obese mice improved insulin resistance and modulated gut microbiota, suggesting a prebiotic-like effect [4]. Zhao et al. [2] concluded that Amelanchier berry powder supplementation in high fat-high sucrose diet-induced insulin-resistant mice dose-dependently attenuated metabolic and inflammatory disorders, which was associated with the amelioration of gut dysbiosis in the mice. Although numerous studies have documented the bioactive and health-promoting properties of *Amelanchier alnifolia* fruits, there is a noticeable lack of data regarding the prebiotic potential of its leaves.

Recent studies have successfully developed delivery systems for plant extracts in combination with prebiotic substances, demonstrating potential benefits for gut microbiota modulation. One notable example is the *Cornus mas* L. lyophilized extract, where the addition of inulin enhances its prebiotic potential by positively influencing the intestinal microbiome [78]. Another study explored *Cannabis sativa* L. extracts combined with dextran, inulin, and trehalose as carriers [79]. These formulations not only supported the growth of beneficial gut bacteria but also preserved antioxidant and neuroprotective properties. Additionally, zein has been investigated as an effective carrier in hesperidin delivery systems, showing improved prebiotic potential [80]. In a system incorporating leaf extracts, the evaluation of dextran-based carriers for Haskap berry leaf extract demonstrated a significant increase in beneficial bacterial strains, particularly *Bifidobacterium longum* (from 9.54 × 10^7^ to 1.57 × 10^10^ CFU/mL) and *Ligilactobacillus salivarius* (from 1.36 × 10^9^ to 1.62 × 10^10^ CFU/mL) [81]. These findings highlight the potential of such delivery systems in shaping gut microbiota composition and promoting overall gut health. However, most studies focus on the activity of the prebiotic substance, and in the case of our study, the extract has a key role in the prebiotic activity of the system and increases it.

The key outcomes of this study, together with their mechanistic explanations, are summarized in Table 7. The table integrates the optimized extraction conditions, the variability of phenolic content and biological activities among cultivars, the results of PCA, FT-IR analysis, release behavior, and prebiotic effects.

## 4. Materials and Methods

### 4.1. Optimization of Extraction

#### 4.1.1. Plackett–Burman Design

Statistical analysis of the experimental data was conducted using Statistica 13.3 software (TIBCO Software Inc., Palo Alto, CA, USA). The Plackett–Burman design was applied to identify the most significant factors influencing the extraction efficiency. The following seven factors were included in the experimental design, each tested at two levels (lower and upper), as in Table 8. A plan of the experiment is illustrated in Table 9.

Dried and powdered leaves of *A. alnifolia* were subjected to extraction under the conditions determined by the Plackett–Burman experimental matrix. The extraction solvent consisted of methanol in varying concentrations, and the process was conducted at different temperatures and pH levels, as defined in the experimental design. Sonication was employed to enhance the extraction efficiency, with varying intensities, to explore its effect on compound recovery. The solvent-to-leaf ratio and extraction time were adjusted according to the design matrix. After the extraction process, the resulting solutions were centrifuged at 4000 rpm for 4 min to ensure complete separation of fine particles. The supernatant was collected and analyzed to determine the total polyphenol content (TPC) using the Folin–Ciocalteu method, with results expressed as mg of gallic acid equivalents per gram of dry weight (mg GAE/g DW).

#### 4.1.2. Box–Behnken Design

The extraction process was optimized using the Box–Behnken design based on factors selected through the Plackett–Burman screening method Statistica 13.3 software (TIBCO Software Inc., Palo Alto, CA, USA). The key variables chosen were methanol concentration in the extraction mixture, the ratio of raw material mass to solvent volume, and extraction time. These factors were tested at three levels to develop a quadratic model describing their influence on extraction efficiency (Table 10). Response surface plots were prepared to visualize the interactions between variables, and the most favorable extraction parameters were determined based on the model (Table 11).

### 4.2. Cultivars Comparision

In the study, 11 different genotypes of Saskatoon serviceberry (*Amelanchier alnifolia*) registered in the National Register of Varieties were tested. Most of the varieties included in the collection of the Central Research Center for Cultivated Plant Variety Testing—Variety Testing Station in Sulechów and the Experimental Variety Assessment Station in Masłowice (φ = 51°15′, λ = 18°38′, H = 174 m above sea level) were analyzed. The plant material for the phytochemical composition analysis came from the fourth year of plantation use, conducted as part of the Post-registration Variety Testing system. The shrubs of the studied *A. alnifolia* species were planted with a row spacing of 300 cm and 150 cm between plants within the row. The experimental field soil belonged to the “good rye complex” of agricultural soil utility, soil quality class IV. The soil type was classified as leached or acidic brown soil with a light loamy sand texture. Soil phosphorus content was at a very high level, magnesium was at a medium level, and potassium was at a low level. The average annual daily air temperature was 8.7 °C, and the total yearly precipitation was 542 mm. In terms of agrotechnical evaluation, the testing of 11 *A. alnifolia* varieties should be considered as an assessment of the biological progress of the studied species, providing objective information on the utility value of the tested varieties and helping farmers make an informed choice of the most valuable genotypes adapted to local cultivation conditions. Table 12 presents a comparative overview of the morphological and organoleptic characteristics of the eleven tested *A. alnifolia* cultivars, including growth vigor, plant habit, root sucker formation, fruit size and shape, wax bloom presence, skin color, sweetness, acidity, and ripening time.

### 4.3. HPLC (High-Performance Liquid Chromatography) Analysis

In order to identify and quantify the major phenolic constituents present in the *A. alnifolia* leaves extracts, high-performance liquid chromatography (HPLC-DAD, LC-2050C, Shimadzu Corp., Kyoto, Japan) analysis was conducted. Separations were performed on a LiChrospher RP-18 column, 5 μm particle size, 250 mm × 4 mm (Merck, Darmstadt, Germany). The detection was performed with a diode array detector at a wavelength maxima (λmax) of 270 and 330 nm, depending on active compounds. The mobile phase was composed of formic acid 0.1% in water (A) and acetonitrile (B) with a gradient elution: 0–35 min; 2–20% B; 35–55 min. 20–70% B; 55 min. 2% B; 55–60 min. 2% B, with mobile phase flow set at 1.0 mL/min. The column temperature was kept at 30 °C.

### 4.4. Antioxidant Activity

#### 4.4.1. DPPH Assay

The antioxidant activity of the samples was determined using the DPPH (2,2-diphenyl-1-picrylhydrazyl) radical scavenging method on 96-well plate. A solution of DPPH (0.1 mM) in methanol was prepared, and 175 µL of this solution was mixed with 25 µL of the sample at various concentrations. The mixture was incubated in the dark at room temperature for 30 min. Absorbance was measured at 517 nm (Multiskan GO, Thermo Scientific, Vantaa, Finland). The percentage of DPPH scavenging was calculated, and IC_50_ values were determined using regression analysis. All measurements were performed in triplicate (*n* = 3).

#### 4.4.2. CUPRAC Assay

The cupric reducing antioxidant capacity (CUPRAC) was assessed by mixing 150 µL CUPRAC reagent (same amount of copper (II) chloride solution (10 mM), ammonium acetate buffer (pH 7.0), and neocuproine solution (7.5 mM)) with 50 µL of the sample. The mixture was incubated for 30 min at room temperature, and absorbance was measured at 450 nm (Multiskan GO, Thermo Scientific, Vantaa, Finland). IC_0.5_ values were determined using regression analysis. All measurements were performed in triplicate (*n* = 3).

### 4.5. Antidiabetic Activity

The α-glucosidase inhibitory activity was assessed using p-nitrophenyl-α-D-glucopyranoside (pNPG) as a substrate. Extracts were prepared in various concentrations and mixed with the enzyme solution in a 96-well plate. After incubation at 37 °C, the substrate was added to initiate the reaction, which was stopped with sodium carbonate. Absorbance at 405 nm was measured to evaluate enzyme activity (Multiskan GO, Thermo Scientific, Vantaa, Finland). The percentage inhibition was calculated by comparing the extract-treated samples to controls, and the IC_50_ value was determined through regression analysis, identifying the concentration required to inhibit 50% of the enzyme activity. All measurements were performed in triplicate (*n* = 3).

### 4.6. Statistical Analysis

Statistical analysis was conducted using Statistica 13.3 (TIBCO Software Inc., Palo Alto, CA, USA). The Shapiro–Wilk test was applied to assess the normality of the data. Differences between mean values were evaluated using ANOVA, followed by Tukey’s post hoc test for multiple comparisons. Statistical significance was set at *p* < 0.05. Correlation analysis was performed using principal component analysis (PCA) and PQStat Software version 1.8.4.142 (2022) (PQStat Software, Poznań, Poland).

### 4.7. System Preparation

The extract from the variety exhibiting the most favorable properties and the highest content of active compounds was obtained under conditions determined by the extraction optimization process. The extract was lyophilized and subsequently dissolved in water together with oligosaccharides—FOS (Sigma-Aldrich, St. Louis, MO, USA), GOS (Chemat, Gdańsk, Poland), and COS (Polaura, Warsaw, Poland)—at a 1:1 (*w*/*w*) ratio. The mixture was stirred for 24 h. The resulting solution was poured onto trays and frozen at −30 °C overnight. Lyophilisation was performed using a freeze-dryer (Telstar, Eden Prairie, MN, USA) for 5 days at −85 °C and a constant pressure of 0.2 MPa. The resulting lyophilizates were then milled using an IKA analytical mill (IKA-Werke GmbH and Co. KG, Staufen, Germany) to obtain homogeneous, free-flowing powders. All systems were stored in tightly sealed Falcon tubes at 8 °C until analysis.

### 4.8. FT-IR Analysis

The (ATR-FTIR) spectra were recorded on an IRTracer-100 spectrophotometer (Shimadzu Corp., Kyoto, Japan). The spectra were measured within the frequency range of 4000 and 400 cm^−1^ in the absorbance mode. The spectral region between 1800 and 2400 cm^−1^ was not included in the main figures, as it does not contain characteristic absorption bands of the analyzed polyphenols and oligosaccharides and is dominated by background signals from atmospheric CO_2_, which could obscure the interpretation of relevant peaks. The parameters of the apparatus were as follows: resolution, 4 cm^−1^; number of scans, 100; apodization, Happ-Genzel. The samples were placed on the ATR crystal and pressed against it whilst the ATR-FT-IR spectrum was scanned. The spectra of pure lyophilizate and oligosaccharides and the physical mixtures of the systems were also analyzed. The spectra were analyzed using the OriginPro 8 software (OriginLab Corporation, Northampton, MA, USA).

### 4.9. PAMPA Assay

Gastrointestinal permeability through passive diffusion was evaluated using a parallel artificial membrane permeability assay (PAMPA) (Pion Inc. Billerica, MA, USA). Samples of GOS, COS, and FOS systems with extract were prepared as described in Section 4.7. Each well of the donor plate was filled with a sample mixed with a donor solution adjusted to pH 6.8. An Acceptor Sink Buffer was added to the wells of the acceptor plate. The microfilter disks separating the donor and acceptor plates were impregnated with GIT lipid to simulate gastrointestinal conditions. The donor and acceptor plates were assembled into a PAMPA sandwich and incubated for 3 h at 37 °C. After incubation, concentrations of active compounds in the donor and acceptor wells were analyzed using a validated HPLC method. The apparent permeability coefficient was calculated using the following equations:

(1) $$P_{app}=\frac{-ln\left(1-\frac{C_{A}}{C_{equilibrium}}\right)}{S\times \left(\frac{1}{V_{D}}+\frac{1}{V_{A}}\right)\times t}$$

where *P_app_* is the effective permeability coefficient (cm/s), *V_D_*—donor volume, *V_A_*—acceptor volume, *C_equilibrium_*—equilibrium concentration, *S*—membrane area, and *t*—incubation time (in seconds). Compounds with *P_app_* < 1 × 10^−6^ cm/s are classified as having low permeability, and those with *P_app_* > 1 × 10^−6^ cm/s as highly permeable compounds. Samples were analyzed in triplicate.

### 4.10. Dissolution Study

Dissolution-rate studies were conducted using a paddle-equipped apparatus (Agilent, Santa Clara, CA, USA). Samples, including 50 mg of the formulated system or pure extract, were placed in gelatin capsules and secured with a sinker to prevent floating. The dissolution media consisted of 0.1 mol/L hydrochloric acid (pH ~1.2) and a solution of phosphate buffer (pH 6.8). The paddles rotated at 50 rpm, maintaining a temperature of 37 °C. At designated time intervals, a 2 mL sample was withdrawn and immediately replaced with a preheated dissolution medium to maintain consistent conditions. The samples were then filtered through a 0.22 μm nylon syringe filter. The dissolved amount of chlorogenic acid, identified as the most abundant compound, was quantified using a validated HPLC method.

### 4.11. Prebiotic Activity

The prebiotic effect of pure lyophilisate Amelanchier leaf extracts, FOS, GOS, COS, and the obtained systems was estimated by measuring the growth of probiotic strains of *Lacticaseibacillus rhamnosus* GG ATCC 53103, *Lactiplantibacillus plantarum* 299v, *Lacticaseibacillus acidophilus* LA-5, *Bifidobacterium longum* DSM 20219 and *Bifidobacterium animalis* DCM 10140 and then calculating the probiotic index value (Ipreb). The effects on pathogenic *Escherichia coli* serotype O157:H7 strain ATCC 43888 were also determined by quantifying the prebiotic activity scores (PAS).

*Lactobacillus*, *Bifidobacterium,* and *E. coli* strains were cultured in MRS broth (Oxoid), MRS broth (Oxoid) with 0.5% (*w*/*v*) cysteine hydrochloride monohydrate (Merck), and Nutrient Broth (Oxoid), respectively. In the above-mentioned media (controls), the carbon source was glucose. To determine the prebiotic effect, 0.1% (*w*/*v*) test materials, i.e., pure extract, prebiotics such as GOS, FOS, and COS, and developed systems were added to these media. All media were inoculated with about 106 CFU/mL with 18–24 h cultures (cultures in the logarithmic phase of growth) and incubated under anaerobic conditions at 37 °C for 24 h. Before (0 h) and after incubation (24 h), the viable cell count was determined using the Koch’s plate method. The solvent for dilutions was physiological saline. Diluted samples were plated on Petri dishes with MRS agar for *Lactobacillus*, MRS agar with cysteine hydrochloride monohydrate for *Bifidobacterium* and Nutrient agar for *E. coli,* and incubated at 37 °C for 48–72 h. After incubation, the grown colonies were counted using an automatic colony counter (Easy Count 2). Based on these, the number of viable cells was determined and given as colony-forming units (CFU/mL).

The results of CFU determinations were used to calculate the prebiotic index from the below equation:

(2) $$Ipreb=\frac{CFU\;of\;probiotics\;in\;prebiotic\;carbohydrate}{CFU\;of\;probiotics\;in\;control\;carbohydrate}$$

In this study the prebiotic carbohydrate were pure extract, FOS, GOS, COS and their systems. While the control carbohydrate was glucose. Prebiotic index higher than 1 is an indication that the carbohydrate has a positive effect on probiotic microbial growth. The prebiotic index is close to 1 shows a poor prebiotic effect of the carbohydrate under study.

Prebiotic activity scores (*PAS*) were also determined using the equation:

(3) $$PAS=\frac{(Log\;P24-Log\;P0)\;prebiotic}{(Log\;P24-Log\;P0)\;glucose}-\frac{(Log\;E24-Log\;E0)\;prebiotic}{(Log\;E24-Log\;E0)\;glucose}$$

where *Log* P are the log of growth (CFU/mL) of the probiotic bacteria at 24 h (P24) and 0 h (P0) in media with prebiotics and glucose; *Log* E are the log of growth (CFU/mL) of pathogenic *E. coli* 24 h (E24) and 0 h (E0) in media with prebiotic and glucose. Positive values PAS indicate a the selective action of prebiotic and its beneficial effect on probiotic growth.

## 5. Conclusions

This study aimed to develop bifunctional systems from *Amelanchier alnifolia* leaves combined with prebiotic oligosaccharides to enhance antidiabetic and prebiotic effects. Extraction parameters were optimized using Plackett–Burman and Box–Behnken designs, establishing methanol content of 58.06%, ratio of 26.03 *m*/*v*, and extraction time of 73.56 min as optimal conditions. Among eleven cultivars, W2 (Obelisk) exhibited the highest polyphenol content and strong antioxidant and α-glucosidase inhibitory activity. Lyophilized systems with FOS, GOS, and COS preserved the bioactive profile, with COS particularly improving chlorogenic acid solubility and modifying its release. FT-IR confirmed intermolecular interactions between polyphenols and oligosaccharides. Prebiotic assays showed a marked stimulation of *B. longum* and *B. animalis*, with multi-component systems achieving prebiotic index values up to twice those of the pure extract. These findings demonstrate that *A. alnifolia* leaf–oligosaccharide systems have significant potential as functional ingredients with dual prebiotic and antidiabetic benefits, warranting further in vivo validation.

## Figures and Tables

**Table 1:** Optimal parameters based on the model.

Parameter	Value
Methanol content (%)	58.06
Ratio (*m*/*v*)	26.03
Time (min)	73.56

**Table 2:** Content of active compounds in different cultivars od *A. alnifolia.* Results are expressed in mg/g DW. The content of the same bioactive compounds was compared across different cultivars, and results marked with different letters indicate statistically significant differences (*p* < 0.05).

	Chlorogenic Acid	Epicatechin	EGCG	Rutin	Hyperoside	Isoquercetin
	(mg/g DW)	(mg/g DW)	(mg/g DW)	(mg/g DW)	(mg/g DW)	(mg/g DW)
W1	8.03 ± 0.01 ^a^	3.48 ± 0.19 ^e,f^	0.98 ± 0.12 ^b^	1.03 ± 0.10 ^c^	2.21 ± 2.28 ^b^	1.65 ± 0.09 ^c^
W2	18.48 ± 0.47 ^f^	3.91 ± 0.09 ^f,g^	1.12 ± 0.01 ^b^	2.28 ± 0.02 ^f,g^	5.77 ± 5.72 ^e^	2.50 ± 0.03 ^d,e^
W3	12.85 ± 0.15 ^c^	1.83 ± 0.02 ^a,b^	2.04 ± 0.01 ^e^	2.02 ± 0.16 ^e,f^	6.08 ± 6.04 ^e,f^	3.39 ± 0.03 ^g^
W4	15.77 ± 0.66 ^d,e^	3.01 ± 0.50 ^e,f^	1.17 ± 0.16 ^b^	2.40 ± 0.18 ^g,h^	5.60 ± 5.76 ^e^	2.65 ± 0.13 ^e,f^
W5	9.97 ± 0.41 ^b^	2.22 ± 0.01 ^b,c^	1.96 ± 0.01 ^d,b^	1.41 ± 0.09 ^d^	3.73 ± 3.46 ^c^	1.27 ± 0.06 ^b^
W6	15.12 ± 0.41 ^d^	4.88 ± 0.08 ^h^	2.55 ± 0.07 ^f^	3.43 ± 0.04 ^i^	4.73 ± 4.78 ^d^	2.84 ± 0.04 ^f^
W7	16.65 ± 0.37 ^e^	2.69 ± 0.10 ^c,d^	1.52 ± 0.01 ^c^	2.44 ± 0.02 ^g,h^	4.08 ± 4.18 ^c,d^	2.27 ± 0.02 ^d^
W8	13.51 ± 0.05 ^c^	4.50 ± 0.03 ^g,h^	3.30 ± 0.03 ^g^	1.72 ± 0.04 ^d,e^	3.52 ± 3.59 ^c^	1.42 ± 0.01 ^b,c^
W9	19.38 ± 0.38 ^f^	2.00 ± 0.13 ^a,b,c^	1.50 ± 0.01 ^c^	0.66 ± 0.04 ^b^	1.89 ± 1.85 ^b^	1.13 ± 0.00 ^b^
W10	8.03 ± 0.01 ^a^	1.40 ± 0.00 ^a^	0.49 ± 0.00 ^a^	0.27 ± 0.01 ^a^	0.89 ± 0.99 ^b^	0.74 ± 0.13 ^a^
W11	12.99 ± 0.11 ^c^	2.07 ± 0.10 ^a,b,c^	1.73 ± 0.02 ^c,d^	2.68 ± 0.01 ^h^	6.35 ± 6.59 ^f^	3.65 ± 0.15 ^g^

**Table 3:** Content of active compounds in the pure extract and in the obtained systems. Values marked with different superscript letters within the same column indicate statistically significant differences (*p* < 0.05) according to one-way ANOVA followed by Tukey’s post hoc test.

	Chlorogenic Acid	Epicatechin	EGCG	Rutin	Hyperoside	Isoquercetin
	(mg/g of System)	(mg/g of System)	(mg/g of System)	(mg/g of System)	(mg/g of System)	(mg/g of System)
FOS system	19.48 ± 0.11 ^a^	3.82 ± 0.17 ^a^	2.47 ± 0.15 ^a^	2.92 ± 0.01 ^a^	7.64 ± 0.01 ^a^	2.83 ± 0.15 ^a^
GOS system	19.75 ± 0.25 ^a^	4.16 ± 0.16 ^a^	2.69 ± 0.10 ^a^	3.08 ± 0.01 ^a^	8.35 ± 0.05 ^a^	3.02 ± 0.02 ^a^
COS system	20.13 ± 0.02 ^a^	4.12 ± 0.14 ^a^	2.55 ± 0.18 ^a^	2.92 ± 0.33 ^a^	7.82 ± 0.82 ^a^	2.87 ± 0.23 ^a^
Freeze-dried extract	39.23 ± 0.75 ^b^	6.93 ± 0.16 ^b^	5.15 ± 0.06 ^b^	7.34 ± 2.37 ^b^	15.35 ± 0.09 ^b^	5.71 ± 0.01 ^b^

**Table 4:** Antioxidant activity of pure lyophilizate Amelanchier leaf extracts and of the obtained systems. Values with different superscript letters in the same column differ significantly (*p* < 0.05, one-way ANOVA followed by Tukey’s post hoc test).

Sample	*CUPRAC* IC_0.5_ (mg/mL)	*DPPH* IC_50_ (mg/mL)
FOS system	0.166 ± 0.005 ^c^	0.172 ± 0.008 ^c^
GOS system	0.164 ± 0.002 ^c^	0.169 ± 0.005 ^c^
COS system	0.166 ± 0.001 ^c^	0.174 ± 0.010 ^c^
Freeze-dried extract	0.083 ± 0.002 ^b^	0.085 ± 0.005 ^b^
Ascorbic acid	0.072 ± 0.003 ^a^	0.068 ± 0.005 ^a^

**Table 5:** Antidiabetic activity of pure lyophilizate of Amelanchier leaf extracts and of the obtained systems. Values with different superscript letters differ significantly (*p* < 0.05, one-way ANOVA followed by Tukey’s post hoc test).

Sample	α-Glucosidase Inhibition IC_50_ (mg/mL)
FOS system	0.29 ± 0.02 ^b^
GOS system	0.31 ± 0.03 ^b^
COS system	0.32 ± 0.01 ^b^
Freeze-dried extract	0.15 ± 0.7 ^a^
Acarbose	3.24 ± 0.03 ^c^

**Table 6:** Results of the experimental in vitro permeability test (PAMPA) for systems and pure lyophilizate of Amelanchier leaf extracts.

Sample	P_app_
FOS system	<1 × 10^−6^ cm/s
GOS system	<1 × 10^−6^ cm/s
COS system	<1 × 10^−6^ cm/s
Freeze-dried extract	<1 × 10^−6^ cm/s

**Table 7:** Summary of the main results and mechanistic insights of the developed *A. alnifolia*-based systems.

Category	Key Findings	Mechanistic Insights
Optimized Extraction Parameters	58.06% MeOH; 26.03 *m*/*v*; 73.56 min (Box–Behnken design)	Optimal solvent polarity and extraction time enhanced phenolic solubility and diffusion, maximizing compound recovery.
Polyphenolic Content	W2 (Obelisk) showed the highest TPC and rich in chlorogenic acid.	High chlorogenic acid and phenolic content provided strong radical-scavenging and enzyme-inhibitory potential.
Antioxidant Activity	W2 (Obelisk), W5 (Thiessen), and W6 (Martin) cultivars demonstrated strongest CUPRAC and DPPH activity.	*A. alnifolia* extract rich in polyphenolic compounds neutralize free radicals through hydrogen donation and electron transfer.
Antidiabetic Activity	W1 (Smoky), W2 (Obelisk), W4 (Northline), and W11 (Pembina) extracts showed strongest α-glucosidase inhibition.	*A. alnifolia* extract rich in polyphenolic compounds inhibits α-glucosidase activity, and has the potential to slow carbohydrate hydrolysis and glucose release.
PCA	PCA explained 68.12% of the total variance (PC1: 48.19%, PC2: 19.93%). Antioxidant capacity (CUPRAC, DPPH), TPC, and chlorogenic acid strongly correlated with PC1. Rutin, hyperoside, epicatechin, EGCG, isoquercetin and α-glucosidase contributed to PC2 differentiation.	PCA confirmed that higher phenolic content, particularly chlorogenic acid, underlies stronger antioxidant activity, and enzyme inhibition is correlated with other flavonoids.
System Preparation	Lyophilized systems maintained bioactive compound content and preserved antioxidant and antidiabetic properties.	Lyophilization avoided thermal degradation, maintaining structural integrity and preserving functional properties.
Molecular Interactions (FT-IR)	FT-IR confirmed hydrogen bonding and interactions with oligosaccharides.	Intermolecular bonds stabilized polyphenols, enhancing solubility.
Chlorogenic Acid Release	The COS system provided the fastest initial release, while the lyophilized extract exhibited the most sustained profile. FOS and GOS systems showed intermediate release patterns.	COS improved solubility via hydrogen bonding, while lyophilization modulated release through matrix entrapment.
Prebiotic Effects	Systems increased PI up to 2×, especially stimulating *B. longum* and *B. animalis.*	Synergy between oligosaccharides and *A. alnifolia* extract promoted beneficial bacterial growth and enhanced prebiotic function.

**Table 8:** Minimum and maximum levels of tested parameters in Plackett–Burman design.

Parameter	Minimum Level (−1)	Maximum Level (1)
Methanol content (%)	0	100
Temperature (°C)	25	80
Time (min)	15	90
Ratio (mg/mL)	10	50
Sonification intensity (%)	0	100
pH	2	10
Particle size (sieve)	125	1000

**Table 9:** Placket–Burman design including seven variables and minimum (−1) and maximum (1) coded values assayed for each.

Run Order	Methanol Content (%)	Temperature (°C)	Time (min)	Ratio (mg/mL)	Sonification Intensity (%)	pH	Particle Size (Sieve)
1	−1	−1	−1	1	1	1	−1
2	1	−1	−1	−1	−1	1	1
3	−1	1	−1	−1	1	−1	1
4	1	1	−1	1	−1	−1	−1
5	−1	−1	1	1	−1	−1	1
6	1	−1	1	−1	1	−1	−1
7	−1	1	1	−1	−1	1	−1
8	1	1	1	1	1	1	1

**Table 10:** Minimum and maximum levels of tested parameters in Box–Behnken design.

Parameter	Minimum Level (−1)	Medium Level (0)	Maximum Level (1)
MeOH content (%)	0	5	100
Time (min)	15	52.5	90
Ratio (mg/mL)	10	30	50

**Table 11:** Box–Behnken design including fifteen variables and minimum (−1), medium (0) and maximum (1) coded values assayed for each.

Run Order	Methanol Content (%)	Ratio (*m*/*v*)	Time (min)
1	−1	−1	0
2	1	−1	0
3	−1	1	0
4	1	1	0
5	−1	0	−1
6	1	0	−1
7	−1	0	1
8	1	0	1
9	0	−1	−1
10	0	1	−1
11	0	−1	1
12	0	1	1
13	0	0	0
14	0	0	0
15	0	0	0

**Table 12:** Comparison of the characteristics of the cultivars.

Variety	Growth Vigor	Habit	Root Suckers	Fruit Size	Fruit Shape	Wax Bloom	Skin Color	Sweetness	Acidity	Ripening Time
Northline (W1)	Strong	spreading	numerous	large	round	present	blue-black	low to medium	high	early to medium
Obelisk (W2)	Strong to very strong	semi-upright	absent or very few	medium	round	present	blue-navy	very high	very low to low	early to medium
Pembina (W3)	Strong	spreading	numerous	medium	round	present	blue-black	medium	medium	early to medium
Sleyt (W4)	Very strong	spreading	medium	medium to large	oval	present	blue-navy	high	low	early to medium
Thiessen (W5)	Medium to strong	upright	few	large	round	present	blue-black	high	medium	early to medium
Honeywood (W6)	Weak to medium	semi-upright	few	medium	round	present	purple-blue	high	low	early to medium
Krasnojarskaya (W7)	Medium	upright	few	large	round	present	blue	high	very low	medium to late
Mandam (W8	Strong	upright	absent or very few	medium	round flattened	present	purple-blue	very high	very low to low	early to medium
Ballerina (W9)	Medium	upright	absent or very few	large	oval	present	purple-blue	high	low	medium to late
BLUE GEM (W10)	Weak to medium	upright	absent or very few	small	round	present	red-purple	low	medium	very early to early
Bluemoon (W11)	weak to medium	upright	few	small	round	present	purple-blue	medium	medium	late

## Data Availability

The data supporting the findings of this study will be made available on the Zenodo repository under the title of the publication.

## Abbreviations

The following abbreviations are used in this manuscript:

COS	Chitooligosaccharides
CUPRAC	Cupric Ion Reducing Antioxidant Capacity
DPPH	2,2-Diphenyl-1-picrylhydrazyl
FOS	Fructo-oligosaccharides
FT-IR	Fourier-Transform Infrared Spectroscopy
GOS	Galactooligosaccharides
HPLC	High-Performance Liquid Chromatography
PAMPA	Parallel Artificial Membrane Permeability Assay
PAS	Prebiotic Activity Score
PI	Prebiotic Index
TPC	Total Phenolic Content
